# Enhancing agro-biodiversity in chicken: a sensory comparison of broths from German local chicken breeds and their crossbreeds

**DOI:** 10.1016/j.psj.2024.103683

**Published:** 2024-03-24

**Authors:** Claire Siebenmorgen, Johanna Mörlein, Micha Strack, Jens Tetens, Daniel Mörlein

**Affiliations:** ⁎ Department of Animal Sciences, Quality of Animal Products, Kellnerweg 6 D- 37077 Göttingen, University of Goettingen, Germany; † ISI GmbH, Rosdorf/Goettingen D-37124, Germany; ‡ Department of Animal Sciences, Functional Breeding, University of Goettingen, Göttingen, Germany

**Keywords:** descriptive analysis, chicken meat quality

## Abstract

The poultry meat production landscape has undergone a reduction in chicken breeds, resulting in a reliance on a limited number of varieties. Motivated by the goal of promoting sustainable chicken production and enhancing agro-biodiversity, this study pioneers a comparison between local chicken breeds (**LB**) and their crossbreeds (**CB**) with modern hybrid lines. Serving as an initial exploration within a larger project, this research acts as a prelude to a comprehensive investigation, aiming to complement the human sensory assessment of product quality. Study I assessed chicken broths prepared from 3 German LBs Bielefelder (**BIE**), Altsteirer (**ALT**), and Ramelsloher (**RAM**) utilizing a factorial 3 × 2 × 2 design that incorporated variations in salt content (unsalted/salted) and cooking time (1 h/3 h). The sensory profiles of the LB broths were largely similar, except for BIE, which exhibited a higher skin odor intensity. Both, increased salt content and longer cooking time intensify sensory perception on most attributes. In study II, BIE was compared with 6 CBs, with variations in salt content and cooking time (6 × 2 × 2 + 1 × 2 factorial design). BIE demonstrated higher sensory intensities than the CBs. Those were comparable, with no clear advantages or disadvantages identified from a sensory standpoint. These findings support that crossbreeding with commercial lines is not associated with changes in the sensory profile. It thus represents a strategy for improving the economic viability of local chicken breeds in order to preserve their valuable agro-biodiversity. The provided protocol for evaluating chicken broth from LBs or their CBs aims to offer researchers a standardized foundation for sensory assessments in chicken broth studies.

## INTRODUCTION

Poultry meat, currently the most produced and consumed globally, is projected to reach nearly 181 million tons by 2050 (Ritchie et al., 2017). As the demand for chicken meat has witnessed a rapid global increase in recent decades, with poultry meat production surging by about 200 % from 45.9 million tons in 1992 to 138.8 million tons in 2022 (FAO, 2022), sustaining this growth becomes a paramount concern. This trend is driven by its unbounded acceptance across dietary practices and its cost-effective, rapid production attributes (Magdelaine et al., 2008). However, the soaring demand in today's poultry production is characterized by strong specialization and has led to a concerning loss of genetic diversity. Due to the characteristic antagonism between laying and meat performance of chicken breeds, hybrid lines that have been intensively bred are primarily used (Augère-Granier, 2019). In 2019, at the Climate Action Summit at the United Nations homogeneity in food was identified as key for lost in diversity (Climate Action Summit, 2019). Additionally, the potential drawback of relying on genetic homogeneity is the vulnerability of these breeds to catastrophes and diseases such as avian influenza. As evidenced by Germany's loss of approx. 400.000 chickens during season 22/23 due to avian influenza (Saechsische Tierseuchenkasse, 2023) this risk underscores the importance of diversifying genetic resources to enhance resilience and mitigate the impact of unforeseen challenges in the future.

Dual-Purpose Breeds (**DPB**) present a promising approach to address the challenges posed by loss of genetic diversity and chick culling in poultry production (Krautwald-Junghanns et al., 2018). Situated at the intersection of natural ecosystems and human-influenced environments, these breeds serve as biodiversity constituents, enhancing agro-biodiversity and positively influencing ecosystem functionality (Sponenberg et al., 2019). Existing literature shows that, while local DPBs frequently exhibit compromised growth performance, feed conversion efficiency, and meat yield in comparison to hybrid lines. There are noteworthy aspects of their meat composition that merit attention: it contains a concentration of nutrients and bioactive components that have substantial benefits for human health and well-being (Charoensin et al., 2021). The literature currently presents an ambiguous conceptualization of DPBs, with variations in interpretation observed both in published works and among practitioners. A concise articulation might suggest that DPBs manifest a balanced ratio between laying and meat performance (Gebhardt, 2023). Due to the absence of a definitive characterization of DPBs in current literature, the breeds under examination herein will henceforth be referred to as Local Breeds (**LB**). Recent research indicates that commercial crossbreeding with modern hybrid lines may be used to improve profitability of the LBs (Jaturasitha et al., 2008; Baldinger and Bussemas, 2021; Escobedo del Bosque et al., 2022). Similar to chicken meat, broths, especially chicken broth, constitute a substantial segment of the global food industry, contributing to a noteworthy market share of 41 % within the USD 5.18 billion worldwide broth market in 2022 (TIP, 2023). The immense popularity and widespread consumption of broths, not only as standalone meals but also as a foundational element in other dishes, underline the need for a thorough sensory evaluation of the product. In the context of poultry, the classic assessment of product quality has traditionally been focused on the juiciness and tenderness of the chicken breast meat – mainly measured with instruments. The human perception actually plays a minor role in scientific papers in terms of the evaluation of poultry meat product quality. It has to be considered, that for 99% of Germans, taste remain the quintessential factor influencing their food choices (BMEL, 2022). Apart from this consumer point of view, the analytical sensory science provides a method to objectively determine the human sensory perception of foods, namely a descriptive analyses (**DA**). Therefore, a complete human sensory evaluation of chickens should start with broths. Broths are obtained from the entire carcass (excluding only the head and feet), which can be evidence of the potential flavor profile of the poultry breed. Given this background, the project OekoGen embarks on an exploration of the potential of 3 LBs and their crossbreeds (**CB**) with 2 commercial origins. The project places particular emphasis on the sensory attributes of the broths derived from these breeds. This focus on broths as the initial step will eventually pave the way for a more extensive evaluation of the meat from these breeds in subsequent studies. Moreover, the study investigates how seasoning broths with salt and varying cooking time influence human sensory perception of the broth. The salting-out effect, induced by the addition of salt, disrupts hydration shells around aromatic substances, allowing flavorings to be released more freely into the broth (Mitchell et al., 2011). An attempt was made to improve the overall flavor profile of the chickens’ broth with a balanced seasoning without overpowering its own taste. In addition to considering the influence of salt, this study also explores the impact of cooking time, particularly within the initial 3 hours period, on enhancing the flavor profile of chicken broth. By examining the relationship between simmering duration and flavor development in chicken broth, valuable insights into the sensory attributes of the broth can be gained (Krasnow et al., 2012; Pérez-Palacios et al., 2017; Qi et al., 2017).

In addition to examining cooking time and seasoning, the main objectives of the study were:

1. To define a sensory profile of chicken broth.
2. To find out whether the 3 LBs differed from one another in sensory terms.
3. To compare sensory profiles between Bielefelder chicken from Study I and 6 crossbreeds from Study II.

## MATERIAL AND METHODS

### Selection of the Animals

#### Pure Breeds

In the project phase, conducted between January 2023 and July 2023, 3 male LBs of local significance: Altsteirer (**ALT**), Bielefelder Kennhuhn (**BIE**), and Ramelsloher (**RAM**) and their 6 CBs with the female modern hybrid lines Lohmann White Rock (**WR**) and Aviagen Ranger (**RG**) were used. The LBs and modern hybrid lines were chosen due to their performance in a preceding trial. The studies were carried out in accordance with relevant national and international guidelines under the EU Directive 63/2010. The animal trial was approved by the local authorities of Lower Saxony under file number 33.19-42502-04-00-00204. Good Veterinary Practice was applied to all procedures whenever animals were handled. The 3 LBs were generated from a parental population by the Poultry Science Working Group at the Institute of Agricultural Engineering at the University of Bonn, Germany and hatched at the Institute for Animal Welfare and Animal Husbandry of the Friedrich-Loeffler-Institute (Celle, Germany). After hatching the one-day-old chicks were transferred to the Institute for Farm Animal Genetics of the Friedrich-Loeffler-Institute (Mariensee, Germany) and were provided with the same commercial starter feed (11.4 Megajoule metabolizable energy/kg dry matter) until they reached 18 d of age. Upon reaching the third week of life, the chickens were transferred to the aviary housing facilities within the Department of Animal Science at the University of Goettingen, Germany. They were housed in groups, with each breed accommodated in separate small groups consisting of up to 15 animals. Throughout the period leading up to their slaughter at 14 wks of age in the slaughterhouse of the University of Goettingen, the animals were fed a commercially available organic feed (Curo GM II, Curo Spezialfutter GmbH, Ennigerloh, Germany). The chickens were stunned according to EU regulations during slaughter, and then their blood was drained through a puncture. Afterward, they were boiled in a 60°C water bath for 15 s, taken out, professionally gutted and weighed (*M* = 1,279 g). Following the slaughter, all animals underwent a chilling process at a temperature of 4°C for a duration of 24 h. After this initial chilling period, the animals were then vacuum frozen in order to minimize possible water loss and stored at a temperature of −18°C for a period of 2 mo.

#### Crossbreeds

Day-old chickens of WR were provided by Lohmann Breeders GmbH (Cuxhaven, Germany) and day-old chickens of RG were provided by Aviagen EW Group GmbH (Visbek, Germany). The chicks were nurtured until sexual maturity at the Frankenforst experimental farm (Koenigswinter, Germany). Crossbreeding was sequentially conducted by integrating 77 individuals of the RG with 11 LBs of each breed (ALT, BIE, RAM). Similarly, 129 individuals of the WR breed were crossed with 19 LBs. The following CBs were established (♂ x ♀): Altsteirer x White Rock (**AWR**), Bielefelder x White Rock (**BWR**), Ramelsloher x White Rock (**RWR**), Altsteirer x Ranger (**ARG**), Bielefelder x Ranger (**BRG**), and Ramelsloher x Ranger (**RRG**). The hatching eggs were collected, transferred and incubated at the Institute for Animal Welfare and Animal Husbandry (Friedrich-Loeffler-Institute, Celle, Germany). Subsequent to this period of incubation, they were moved to the Gut Merbitz GbR (Wettin-Loebejuen, Germany), after hatching as day-old chicks. The chickens were fed with an organic starter feed (14.2 megajoule metabolizable energy/kg dry matter) until 5 wk of age and Grower I feed (13.7 megajoule metabolizable energy/kg dry matter) until 5 wk of age (WS 1-Gefluegelhof Schubert, Unterruesselbach, Germany). From 5 wk of age until slaughter, the chickens were fed Grower II feed (13.6 megajoule metabolizable energy/kg dry matter). During the initial phase of their life, up to their 10th wk, these male animals were kept in relatively large groups (225 to 258 animals). From the 10th week onwards, they were segregated into smaller groups (22 animals). Upon 12 wk of age, the animals were transported to the Bioland Roth slaughterhouse (Witzenhausen, Germany), where they were processed. In compliance with EU regulations, the chickens were electrically stunned in a water bath and subsequently bled out through a puncture. Following this, the birds underwent a 12 s boiling process in a 60°C water bath. They were then extracted, professionally gutted and weighed (*M* = 880 g). The CBs were vacuum packed 24 h after slaughter and kept at 4°C for a week.

### Panel Screening and Training

According to ISO 8586, a descriptive analysis involves extensive training of panelists that enables the panelists to characterize the flavor notes and their intensities using scales. The sensory panel for this study comprised of 11 panelists, screened based on their individual olfactory and gustatory performance out of 40 persons. Both oral and written informed consents were obtained. The sensory screening, training and analysis took place in the sensory laboratory of the Agricultural Faculty of the University of Goettingen according to ISO 8589 (ISO, 2010) under ethical clearance. Panelists were compensated with 17 € per h for their participation. Due to significant inter-individual variations in sensitivity, specific taste tests were conducted for panel screening. Basic taste identification was conducted with the tastes sucrose (sweet), sodium chloride (salty), citric acid (sour), caffeine (bitter), and monosodium glutamate (umami) at the concentration levels as per DIN 8586 (ISO, 2014) and an elevated concentration of an additional 30%. After screening the selected panelists were trained for odor identification performance (multiple choice task) based on the panel training proposal from Trautmann et al. 2014. A total of 12 fragrance strips, each with a concentration of 1.5%, were produced using the following procedure: 15 µL of liquid substances were pipetted into 985 µL of propanediol and then vortexed for 30 s. Then 20 µL of the resulting solution was pipetted onto the olfactory strip. The substances were the following: 3-cis-hexenol (fresh gras), 1-menthol (mint), cinnamaldehyde (cinnamon), eugenol (clove), octen-1ol-3 (fungus), γ-undecalaton (peach), d-Carvone (caraway), β-ionone (violet). For androstenone (AN3), 15.9 µl of a solution consisting of 200 µL androstenone and 800 µL methanol (**MeOH**) was mixed with 1,479.6 µL propanediol, followed by the application of 20 µL onto the strips. In the case of skatole (**SK7**), 15.3 µL of a solution containing 100 µL skatole and 900 µL MeOH were mixed with 1,484.7 µL propanediol, and 20 µL of the resulting mixture were applied to the strips. Amino acetophenone (**AAP3**) was prepared by mixing 25.3 µL of a solution comprising 100 µL amino acetophenone and 900 µL MeOH with 1,474.7 µL propanediol, and 20 µL of this mixture were applied to the strips. Additionally, the vanilla strip, with a concentration of 0.05%, was formulated by dissolving 25 mL vanillin in 50 mL propanediol, and 20 µL of this solution were applied to the strips. Also for training a stepwise staircase threshold test according to ISO 3972 (ISO, 2011) for the taste of salty, sweet, and umami was conducted. The results of the panel performance, after 6 training sessions, with each session lasting 2 h, resulting in a cumulative training duration of 12 h, is shown in Table 1.

**Table 1:** Summary of sensory panelist training outcomes after 12-h training: descriptive statistics for 12 olfactory tests (multiple choice) and threshold evaluations (thresholds 1–9) of 3 basic taste types salty, sweet and umami (g/ L tap water).

	Odor identification^1^	Salty threshold^2^	Sweet threshold^2^	Umami threshold^2^
Mean	9.8	5.1 (0.48 g sodium chloride/L)	6.1 (2.59 g sucrose/L)	5.7 (0.24 g monosodium glutamate/L)
Minimum	8	3 (0.24 g sodium chloride/L)	4 (0.94 g sucrose/ L)	4 (0.17 g monosodium glutamate/L)
Maximum	12	9 (2 g sodium chloride/L)	9 (12 g sucrose/L).	9 (1 g monosodium glutamate/L)

1 According to Trautmann et al. (2014).

2 According to ISO (2011).

### Sensory Descriptive Analysis

The Descriptive Analysis (**DA**) was conducted to obtain a detailed qualitative and quantitative sensory profile of chicken broth in terms of the sensory modalities odor and taste. First step in training for DA was to identify specific attributes for chicken broth. To familiarize the panelists with chicken broth, an initial evaluation of 3 h cooked unseasoned chicken broth, beef broth, and pork broth was carried out. The verbalization of the chicken broth's properties and attributes works best in contrast to analogous products. In the following 4 training h various commercial chicken broths (BROX organic chicken bone broth, Potsdam, Germany; URGESCHMACK chicken broth, Großalmerode, Germany; LACROIX chicken stock, Zurich, Switzerland; SONNEN-BASSERMANN chicken stock, Frechen, Germany) were prepared and evaluated by the panelists. Additionally, a commercial organic chicken sourced from a local supermarket and one of the later used target chickens (**BIE**) were cooked in all training sessions and evaluated to elucidate additional attributes. For the BIE sample panelists agreed on reference intensities on a 0 to 100 % intensity scale for each attribute. The final attribute lexicon for chicken broth contained 7 odor and 9 flavor attributes shown in Table 2. In collaboration with the panelists, 8 references and 5 flavor threshold solutions were chosen that optimally represented the 16 attributes and were deemed most appropriate for the evaluation of the broths. The 8 references were presented to the panelists in 50 mL Duran beakers (DWK Life Sciences, Wertheim, Germany). The 5 flavor threshold solutions were presented to the panelists in 25 mL Melipul disposable medication cups (SCHWARZ, Isny, Germany).

**Table 2:** Attributes and corresponding references for sensory evaluating of the classes odor, taste/ aroma, and aftertaste for 1 and 3 h cooked chicken broth of local breeds and their crossbreeds using the whole carcass, presented in order of evaluation.

Class	Attributes	Order of evaluation	References	Amount presendted
Odor	Chicken intensity	1	warm chicken breast meat (stewed in tap water at 89-95°C for 3 h)	15 g
	cabbage-like intensity	2	1 day old 1 h cooked cold cabbage soup (stored overnight at +4°C)	50 mL
	skin intensity	3	warm chicken skin (stewed in tap water at 89-95°C for 3 h)	10 g
	sour intensity	4	fresh juiced lemon in tap water (ratio 1:4)	30 mL
	kokumi intensity	5	korean black bean paste Jjajang (ASSI, Seoul, South Korea)	10 g
	sweet intensity	6	sunflower honey (LAGNESE, Bargteheide, Germany)	10 g
	general intensity	7	warm standardized broth (stewed whole carcass for 3 h in tap water, unsalted broth)	50 mL
Taste/ aroma	Cabbage-like	8	1 day old 1 h cooked cold cabbage soup (stored overnight at +4°C)	50 mL
	chicken	9	warm chicken breast meat (stewed in tap water at 89 to 95°C for 3 h)	15 g
	sweet	10	sucrose solution threshold 6 (2.59 g/L in tap water)	15 mL
	salty	11	stock solution sodium chloride (4.0 g/L in tap water)	15 mL
	sour	12	citric acid solution threshold 7 (0.38 g/L in tap water)	15 mL
			and threshold 5 (0.25 g/L in tap water)	15 mL
	umami	13	stock solution monosodium glutamate (2.0 g/ L in tap water)	15 mL
Aftertaste	Cabbage-like	14	1-day-old 1 h cooked cold cabbage soup (stored overnight at +4°C)	50 mL
	Fatty	15	goose fat (LARU, Bottrop, Germany)	20 g
	umami	16	stock solution monosodium glutamate (2.0 g/ L in tap water)	15 mL

The assessments for study I and study II were performed on separate days to ensure independent assessments. All sessions of study I and II were conducted at 21°C in a sensory lab with 10 booths. The laboratory was equipped with red lights (ISO 8589), which were used to minimize any visual cues related to the appearance of the broth samples, allowing the panelists to focus solely on odor, taste, and aftertaste. All attributes’ intensities were rated using a 15 cm line scale, as recommended (Gomide et al., 2021). The scale ranged from 0 cm, representing low intensity (anchor “0”), to 15 cm, indicating high intensity (anchor “100”) for the attribute being evaluated. Prior to each session, a calibration period of 5 min was allocated at the beginning, during which the panelists familiarized themselves with the references. Subsequently, to minimize potential order-effects, the samples were presented to the panelists in a randomized design. Each sample was blinded by a 3-digit number. The data collection was performed utilizing EyeQuestion software (Version 5.2, Elst, Netherlands).

### Broth Preparation

Frozen LB carcasses, aged 2 mo, were thawed under refrigeration at +4°C for a duration of 24 h. Subsequently, the carcasses were rinsed with cold running water and placed whole in a 20 l stainless steel pot (GSW 643474, Spabrücken, Germany), with a ratio of carcasses to cold water set at 1:4 (water weighing 3 times more than the chicken). By utilizing the whole carcass, we aimed to maximize the flavor and sensory attributes. All pots were positioned on an induction hotplate (IK 35dp, Bartscher, Salzkotten, Germany) and brought to boil at power level 5. After approximately 20 min, a layer of white foam, consisting of proteins, formed on the surface of the broths. This foam was carefully skimmed off and discarded. Following boiling at a temperature range of 100 to 105°C, the heat level was reduced to level 1, enabling the broths to simmer slowly within the temperature range of 89 to 95°C, to reduce steaming loss. After 1 h of simmering, 500 mL of each of the 3 broths were transferred to 500 mL Duran beakers (DWK Life Sciences, Wertheim, Germany). The top layer of oil was removed through skimming. Subsequently, the broth samples were labeled with respective 3-digit codes and transferred to 50 mL Duran beakers for cooling.

In session 2, the broths underwent supplementation with 0.5 g of sodium chloride per 100 mL of broth. This salt concentration was derived from the concept of a low salted broth, as outlined by Mitchell et al. (2011). Utilizing a weakly salted broth was to ensure that the salt did not overpower the intrinsic taste of the broth itself. However, it was also intended to facilitate the release of certain flavor compounds through the “salting-out” effect. The remaining broths were further simmered for an additional 2 h and were also salted with 0.5 g of sodium chloride per 100 mL of broth in session 2. At the end of the 3 h cooking period, another 500 mL of each broth were taken out, the oil layer was skimmed off, and the broths were transferred to another set of 50 mL Duran beakers. Both sets of beakers, representing the 1 h and 3 h cooking sessions, were then placed on trays and placed in oven at a temperature of 80°C for a minimum of 10 min using a steam cooker (Hansdampf Autochef Konvektomat, MKN, Wolfenbüttel, Germany). This was done, in order that the panelists could evaluate all the sample broths at 54 to 60°C as recommended in Qi et al. (2021).

In both sessions of study I, the panelists evaluated 6 samples each (3 breeds x 2 cooking times). For study II, session 1 and 2 followed a similar procedure as study I, with a total of 13 samples per session (6 CBs x cooking times, + BIE as reference). The preparation method for these CBs remained equal to that of the LBs; salt and cooking time varies between sessions, too. Table 3 provides a comprehensive summary of the specific procedures employed in both study I and study II.

**Table 3:** Design for study I and study II of sensory evaluation of chicken broths made from local chicken breeds and their crossbreeds. Evaluation of 1 h or 3 h cooked and salted or unsalted broths were conducted without replicates.

Study number	Session number	Number of panelists	Breed	Cooking time	Seasoning
study I	session 1	11	ALT, BIE, RM	1 h and 3 h	unsalted
	session 2	11	ALT, BIE, RM	1 h and 3 h	salted (0.5 g sodium chloride/ 100 mL tap water)
study II	session 1	11	AWR, BWR, RWR, ARG, BRG, RRG	1 h and 3 h	unsalted
			BIE	3 h	unsalted
	session 2	10	AWR, BWR, RWR, ARG, BRG, RRG	1 h and 3 h	salted (0.5 g sodium chloride/ 100 mL tap water)
			BIE	3 h	salted

Abbbreviations: ALT, Altsteirer; BIE, Bielefelder; RM, Ramelsloher; AWR, Altsteirer x White Rock; BWR, Bielefelder x White Rock; RWR, Ramelsloher x White Rock; ARG, Altsteirer x Ranger; BRG, Bielefelder x Ranger; and RRG, Ramelsloher x Ranger.

### Statistical Analysis

The sensory data were analyzed by a linear mixed model in IBM SPSS Statistics software (v. 28; IMB Corporation, New York, NY). The model incorporated the 3 fixed factors breed, cooking time and salting, and the random factor panelist. Additional to all main effects, 2-way interactions of the fixed factors were included in the design. Statistical significance was set to *p* ≤ 0.05. To visualize the estimated means, a principal component analysis (**PCA**) was conducted. PCA is a statistical technique used to reduce the dimensionality of the attributes while retaining the most relevant information. The PCA was performed in IBM SPSS Statistics based on correlations after standardization.

## RESULTS

### Comparative Analysis of Pure-Bred Animals (Study I)

The 3 × 2 × 2 (breed x salt x cooking time) factorial design of study I for the LBs did not reveal major significant differences between the sensory profiles of the 3 LBs. Only in the odor attribute of skin intensity a significant breed effect was observed (*p* = 0.047). Specifically, the odor skin intensity of the BIE (*M* = 41.2) was significantly higher, compared to ALT (*M* = 37.8), with RM (*M* = 39.2) in between. Additionally, a breed x salt (*p* = 0.006) interaction on the odor skin intensity appeared, showing the BIE advantage especially in the salted condition.

### Comparative Analysis of Crossbreed Animals (Study II)

**Figure 1:**
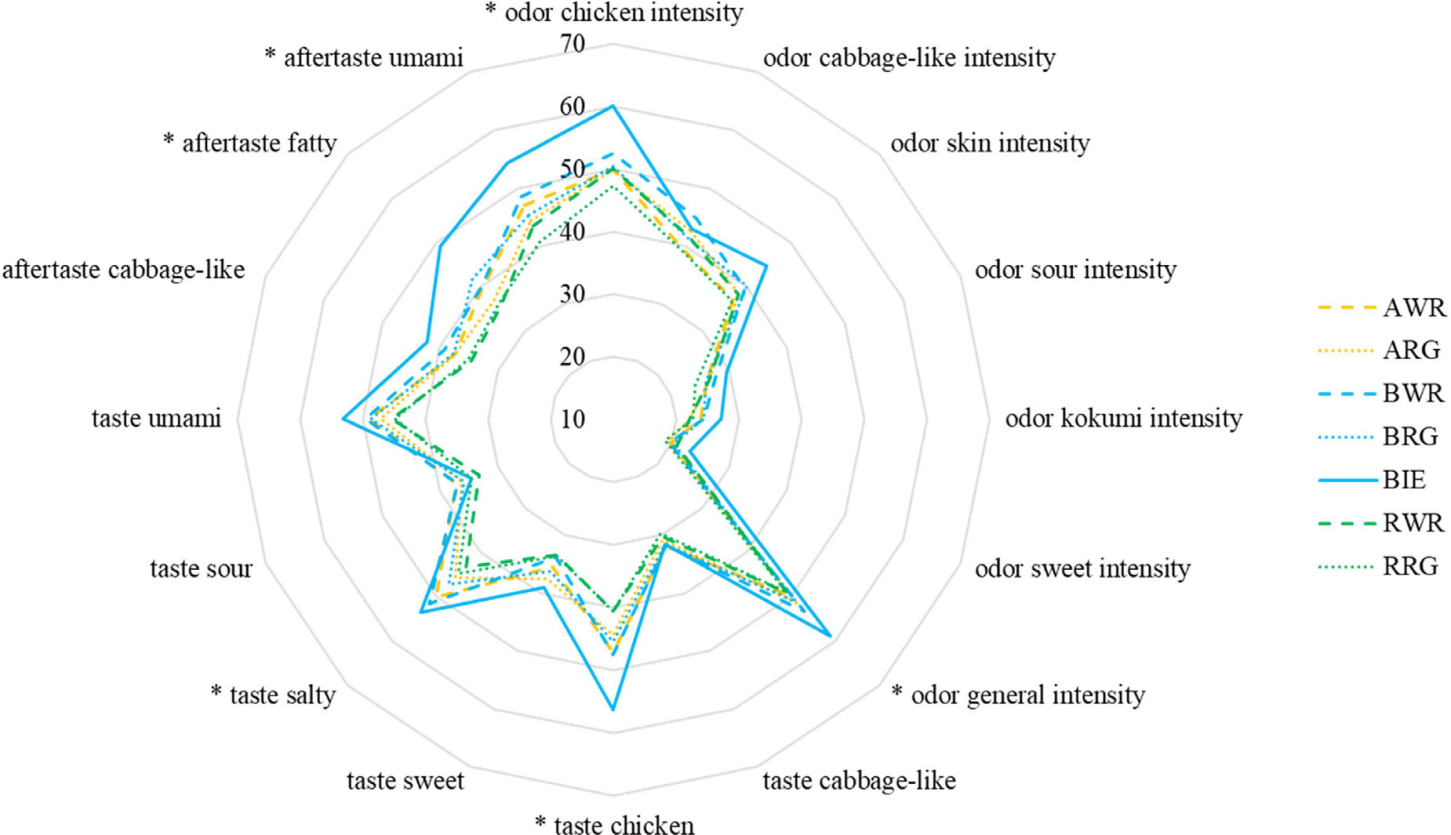
Sensory profiles from study II with 16 attributes for 6 crossbreeds and the local breed Bielefelder. The Bielefelder breed is evaluated significantly more intensive in 6 out of 16 attributes, in comparison to 6 crossbreeds. ARG = Altsteirer x Ranger, AWR = Altsteirer x White Rock, BRG = Bielefelder x Ranger, BWR = Bielefelder x White Rock, BIE = Bielefelder, RRG = Ramelsloher x Ranger, RW = Ramelsloher x White Rock, *: *p* < 0.05.

In the 6 × 2 × 2 + 1 × 2 factorial comparison between the 6 CBs (AWR, ARG, BWR, BRG, RWR, RRG) and additional BIE purebred (3 h, unsalted), significant breed effects were observed in various attributes within the odor and taste modality. The odor of the breed's broth differs in chicken intensity (*p* = 0.014) and general intensity (*p* = 0.028). Breed effected the intensity of chicken meat (*p* = 0.001) and saltiness (*p* = 0.001). Furthermore, the aftertaste differs in fatty (*p* = 0.001) and umami intensity (*p* = 0.001). However, the CBs showed rather no notable sensory differences amongst themselves, whereas the BIE purebred evokes a different sensory profile when compared to the 6 CBs, as depicted in Figure 1. The BIE evokes a significantly more intensive profile in 6 out of 16 attributes.

### Effect of Cooking Time

**Figure 2:**
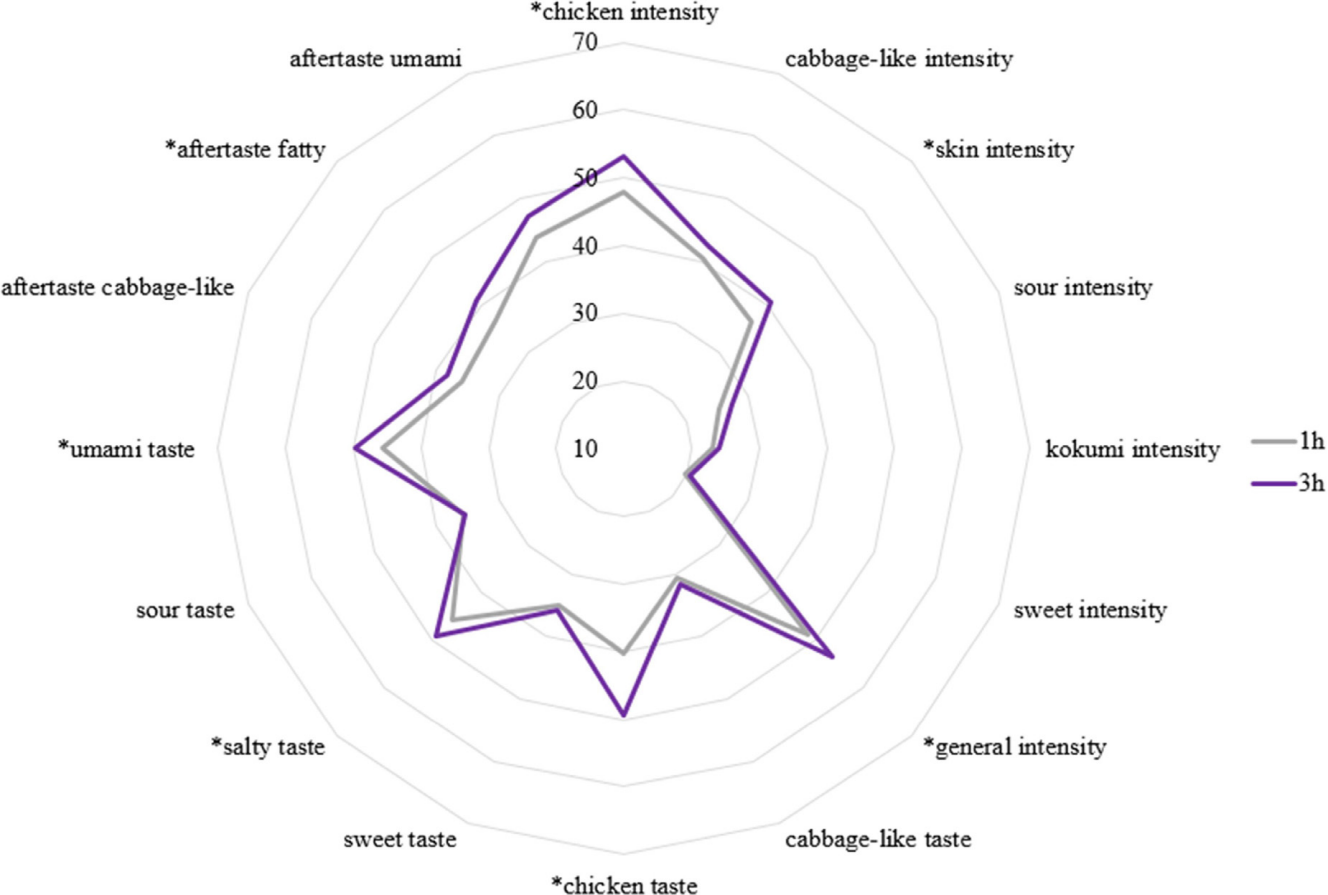
Sensory profiles from study II of chicken broth in the 16 attributes for different cooking time (1 h and 3 h) for 6 crossbreeds and the local breed Bielefelder. The pure breed Bielefelder is evaluated significantly more intensive for 7 out of the 16 attributes, in comparison to 6 crossbreeds, *: *p* < 0.05.

The impact of cooking time on sensory profiles, was found in both study I and study II. Longer cooking time increased the odor intensity in chicken, cabbage-like, skin, kokumi, and general intensity as well as the taste in chicken intensity and salty and the aftertaste of cabbage-like, and umami (study I). The same effect is observed in the CBs (study II). Here, the odor intensifies in chicken intensity, skin and the general intensity; the chicken, salty and umami taste and also the fatty aftertaste (Figure 2).

### Effect of Seasoning (Salted/ Unsalted)

The addition of salt (at a concentration of 0.5 g/100 mL broth) intensifies certain sensory properties of the broth. Specifically, in study I the inclusion of salt enhances the perception of tastes cabbage-like (*p* = 0.002), chicken (*p* = 0.039), salty (*p* = 0.001), umami (*p* = 0.013) and aftertaste umami (*p* = 0.003). Moreover, a compensatory interaction between salting x cooking hours appeared for the taste of chicken (*p* = 0.032), salty (*p* = 0.005), and the fatty aftertaste (*p* = 0.019) (Table A1). Similarly, in study II the addition of salt to the CB broths resulted in a significant enhancement of chicken intensity odor (*p* = 0.001), skin odor (*p* = 0.002), general odor intensity (*p* = 0.001) as well as the chicken taste (*p* = 0.001), salt taste (*p* = 0.031), umami taste (*p* = 0.008) and fatty aftertaste (*p* = 0.035) (Table A2).

**Figure 3:**
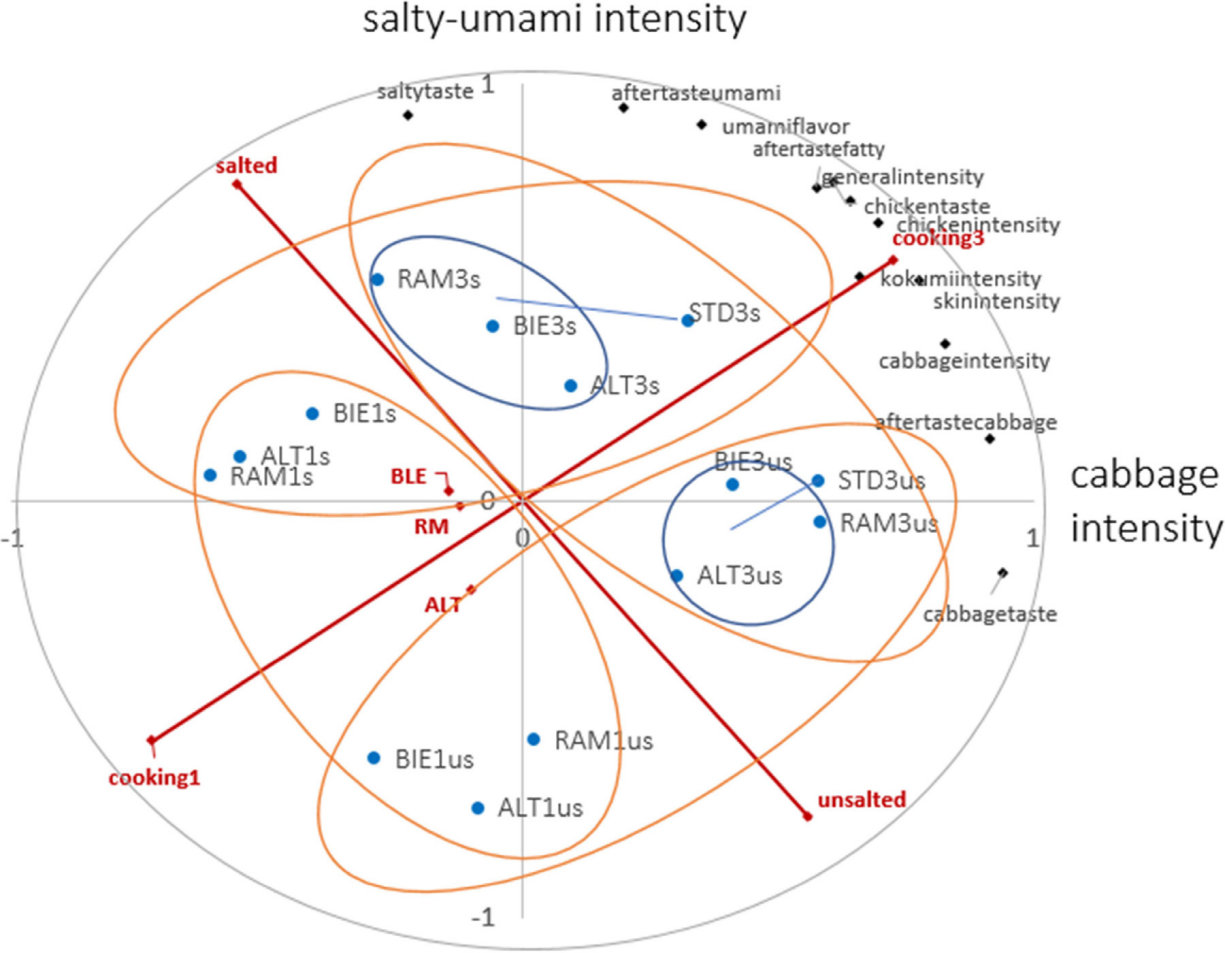
Study I: Correlations of all attributes and factors for chicken broth of 3 local breeds and one Standard. Red: Correlations of factor levels (main effects) with PC of 14 discriminating attributes (black) from 14 samples (blue; estimates from a mixed model with sample as fixed factor. BIE = Bielefelder, ALT = Altsteirer, RAM = Ramelsloher and STD = Standard, 1 and 3 = cooking hours, us = unsalted, s = salted.

**Figure 4:**
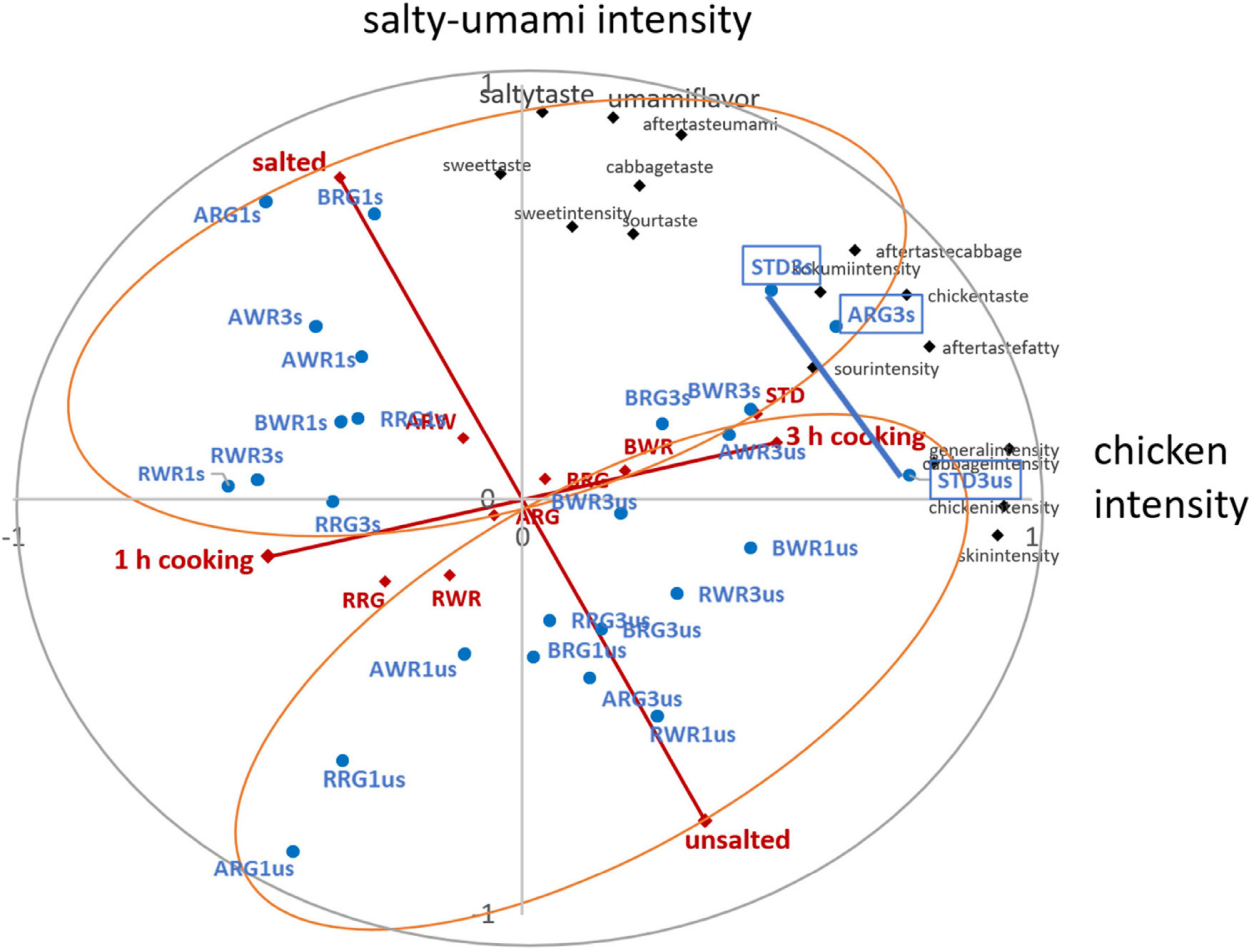
Study II: Correlations of all attributes and factors for chicken broth of 6 crossbreeds and one Standard. Red: Correlations of factor levels (main effects) with PC of 16 attributes (black) from 26 samples (blue; estimates from a mixed model with sample as fixed factor. ARG = Altsteirer x Ranger, AWR = Altsteirer x White Rock, BRG = Bielefelder x Ranger, BWR = Bielefelder x White Rock, RRG = Ramelsloher x Ranger, RW = Ramelsloher x White Rock, STD = Standard, 1 and 3 = cooking hours, us = unsalted, s = salted.

Figures 3 and 4 summarize the results from study I and study II in form of a PCA-map. At a single glance, the 2 strongest main effects can be clearly identified: The inclusion of salt leads to a distinct separation of sensory profiles, with perfectly separated samples representing a shift towards a more salted-umami profile. The graph also reveals that a longer cooking time intensifies the chicken flavor in the CBs. This effect is visually represented by a noticeable expansion or concentration of sensory samples in the chicken flavor region, indicating a clear relationship between cooking time and the intensity of chicken flavor. In addition to these main effects, it can also be observed in study II that the BIE (always 3 h cooked) correlates towards the chicken region as well, implying a similarity to the CBs in the sensory profile.

## DISCUSSION

The objective of this study was to investigate sensory differences of broths made from 3 LBs and their 6 CBs with modern hybrid lines. To the best of our knowledge, there are no descriptive analyses for broths of LBs and their crossbreeds. Few comparable studies focus on characterizing genetic factors of other native LBs or analyzing substance shares in broths rather than solely on sensory evaluation of these broths (Jeon et al., 2010; Fan et al., 2018; Wang et al., 2020).

Based on findings from previous studies, it is assumed that the taste, odor and aroma characteristic of chicken products is relatively complex and is mostly genetically controlled (Zhang et al., 2021). A favorable relationship between chicken breed type and a higher inosine monophosphate content (which has a flavor-enhancing effect) has been demonstrated in native Chinese chicken breeds (Ye et al., 2010). Flavor and quality variations in chicken broths, even from the same breed, can arise due to pre-slaughter factors, such as age, sex, and diet (Zhang et al., 2021). Jayasena et al. (2015) found that as traditional Korean chickens aged, there was a decline in amino acids contributing to the sweet, umami, and bitter taste. Herein the LBs were slaughtered at 14 wk, while the CBs were slaughtered at 12 wk. This difference was mirrored in our findings, wherein ALT and RAM exhibited a significant lower evaluation in the sweet and umami taste compared to the CBs. Drawing from research by Lyon et al. (2004) broilers fed on corn produced breast meat that garnered higher sensory evaluation scores in broths than those fed with milo or wheat. Given that, the LBs and CBs in our research received distinct feed mixtures, the influence of these feeding variations on taste profiles cannot be dismissed.

Another reason why BIE was rated more intensive than the CBs may also be the storage after slaughter. The CBs in the present study were not frozen, unlike the LBs, which were for 2 mo at -18°C. This can lead to different maturation processes in the carcasses, which in turn affects the sensory properties. Qi et al. (2021) showed that short-term deep-freeze storage of raw chicken meat enhances the aroma intensity of the broth. Ice crystals can form and distribute the intra- and extracellular-components of the meat, so more aromatic substances like aldehydes (with higher hydrophobia) and 2-pentylfuran (odor-active compounds) can defund in the prepared broths. On the other hand, Soyer et al. (2010) showed, that prolonged frozen-storage can also have negative effects on the chicken meat, because of increased lipid oxidation, which is the most important factor of quality loss like flavor and nutritive value. A prolonged deep-freeze storage could thus have been an influencing factor in this study.

Besides storage the chicken's preparation and cooking process can greatly impact the broths sensory properties. Critical factors include the meat-to-water ratio, heat treatment (temperature and duration), cooking methods, and spice use all of which substantially dictate chicken broth quality. While other studies on chicken broths used a meat weight:water ratio of 1:1 (Zhan et al., 2020), 1:2 (Rothe et al., 1981; Qi et al., 2017; Qi et al., 2021) or 1:3 (Rothe et al., 1981), the present study used 3 times the water of the chicken weight. This was done so the whole undivided carcass was fully submerged to ensure even coverage. However, this might dilute the broth's intensity as the flavor compounds have more water to disperse into. In addition to the water ratio, the cooking time and temperature of the broth are also decisive. According to Qi et al. (2017) a chicken broth reaches its maximum aroma after 3 h of simmering. In general, the simmering time has a positive effect on improving the taste profile of chicken broth, especially within the first 2 h. Pérez-Palacios et al. (2017) suggested that, when preparing chicken broth, the formation and diffusion of amino acids and nucleotides (aroma compounds) are promoted by a temperature around 100°C and a long cooking time (5 h). It was recommended to cook chicken broths for 3 to 5 h at about 100°C, as there are significant correlations between the content of amino acids and nucleotides and the taste of chicken broths with inosine monophosphate. Because of these results and a limited time span in the mornings of study I and II, we chose the cooking time of 1 h and 3 h. We were able to demonstrate in the present study that the cooking times between 1 h and 3 h significantly influence almost all described attributes of the chicken intensity dimension (*p* < 0.05). As Krasnow et al. (2012) demonstrated, broths prepared at 99°C scored overall better in the sensory evaluation than broths cooked at 85°C. Due to the technical conditions of the movable induction plates in the present study, a constant temperature above 94°C was not possible without the pots boiling over. In future studies, a longer cooking time of 5 h and close to 100°C should be done. Our research demonstrated also that even a minuscule salt concentration significantly intensified several attributes. As outlined by Mitchell et al. (2011) the salting-out effect can promote the dissolution of various aroma compounds. However, the salting-out effect necessitates careful temporal regulation of salting, ideally towards the end of cooking time. Earlier salting could cause the excessive dissolution of flavor compounds into the broth, leading to their volatilization with steam and subsequent loss, thus preventing their accumulation in the broth. Considering these findings, we strictly adhered to end-cooking salting throughout our experimental procedure.

In light of the results demonstrating a significant superiority of the BIE to the other LBs and CBs, it can be inferred that further expansion of the BIE husbandry and conducting additional studies on these animals should be pursued. As a result, biodiversity can be increased and preserved. In total there might be potential to develop a niche market for LBs, but it necessitates the initiative of a competent market party (Leenstra et al., 2010), the formulation of marketing strategies, and the successful persuasion of consumers regarding the benefits. The identification and conservation of LBs are crucial to fulfill unforeseen future breeding requirements. For subsequent investigations focusing on chicken broth, our attribute table (Table 2), formulated through an extended training and attribute identification process with the panelists, offers a robust framework for the DA of chicken broths. The attributes identified within this study contribute significantly to the sensory research repository and serve to facilitate a more comprehensive and streamlined evaluation of poultry.

## CONCLUSIONS

The results of this research show the importance of chicken broth evaluation when determining product quality of poultry. Concluding from descriptive sensory analyses, chicken broths from local breeds (LBs) and from their cross-breeds with commercial lines (**CB**) were similar; no clear advantages or disadvantages could be identified. Both hybrid lines are considered suitable mating partners for crossbreeding. For crossbreeding is not associated with changes in the sensory profile, it represents a great potential to improve biological performance and hence economic viability. Thus, the endangered local breeds of LBs and CBs and their valuable agro-biodiversity of poultry can be preserved. Results prove the positive intensifying effect of an extended cooking time (3 h) on human flavor perception of chicken broths. Adding small amounts of salt (0.5 g/100 mL broth) significantly intensifies the attributes of chicken broths.

## ACKNOWLEDGMENTS

This project was founded by the Federal Ministry of Food and Agriculture (BMEL) based on a decision of the Parliament of the Federal Republic of Germany via the Federal Office for Agriculture and Food (BIE) under the innovation support program (FKZ: 28190E167). We extend our profound gratitude to the entire team of the project OekoGen, as this study would not have materialized without their invaluable contributions. We would also like to thank our loyal panelists, without whom this study would not have been possible. Special thanks to Carina Blaschka and her team for their dedication in rearing and nurturing the LB chickens, and to Margret Krieger and her team for their diligent care of the CB chickens. We also appreciate the expertise and assistance provided in the sensory lab by Steffen Hassenpflug, Ankita Shrestha, and Ruth Wigger. Furthermore, our gratitude is extended to the entire staff at the slaughterhouse for their steadfast support.

Author contributions: Project acquisition: JT, DM. Conceived the sensory study: JM, DM. Supervision: DM, JM, MS. Performed the sensory experiments: JM, CS. Raised the LB-chickens: JT. Organized sample collection: CS. Analyzed the data: JM, CS, MS. Wrote the manuscript: CS, JM. Involved in the discussion of the manuscript: JM, CS, MS, JT, DM. All authors have read and approved the manuscript.

## DISCLOSURES

The authors declare no conflicts of interest.

## Figures and Tables

**Table A1:** Study I: *P*-values of the 3 × 2 × 2 factorial comparison (**LMM**) between 3 local breeds (Altsteirer, Bielefelder, Ramelsloher), seasoning (salted/ unsalted) and cooking hours (1 h/3 h), as quantified by 15 cm line scales (0–100).

Class	Attributes / *p*-value	Breed	Salt	Hours	Breed x Salt	Breed x Hours	Salt x Hours
Odor	Chicken intensity	0.440	0.498	**0.001**	0.294	0.304	0.225
	Cabbage-like intensity	0.289	0.447	**0.001**	0.074	0.960	0.623
	Skin intensity	**0.028**	0.998	**0.001**	**0.013**	0.931	0.234
	Sour intensity	n.a	n.a	n.a	n.a	n.a	n.a
	Kokumi intensity	0.669	0.412	**0.001**	0.161	0.528	0.652
	Sweet intensity	0.274	0.334	0.193	0.873	0.208	0.177
	General intensity	0.149	0.167	**0.001**	0.063	0.431	0.477
taste/ aroma	Cabbage-like	0.496	**0.002**	**0.001**	0.901	0.570	0.590
	Chicken	0.115	**0.039**	**0.001**	0.850	0.808	**0.032**
	Sweet	0.977	0.631	0.240	0.126	**0.040**	0.336
	Salty	0.258	**0.001**	**0.001**	0.370	0.251	**0.005**
	Sour	0.887	0.943	0.054	0.690	0.500	0.645
	Umami	0.680	**0.013**	**0.001**	0.951	0.842	0.160
Aftertaste	Cabbage-like	0.444	0.216	**0.002**	0.127	0.831	0.358
	Fatty	0.787	0.184	**0.001**	0.962	0.867	**0.019**
	Umami	0.684	**0.003**	**0.001**	0.699	0.874	0.294

The bold marked values are the ones that are significant.

**Table A2:** Study II: *P*-values of the 6 × 2 × 2 + 1 × 2 factorial comparison (**LMM**) between 6 crossbreeds (Altsteirer x Ranger, Altsteirer x White Rock, Bielefelder x Ranger, Bielefelder x White Rock, Ramelsloher x Ranger, Ramelsloher x White Rock) and Bielefelder purebreed with different seasoning (salted/unsalted) and cooking hours (1 h/3 h), as quantified by 15 cm line scales (0–100).

Class	Attributes / *p*-value	Breed	Salt	Hours	Breed x Salt	Breed x Hours	Salt x Hours
Odor	Chicken intensity	**0.014**	**0.001**	**0.011**	**0.021**	0.142	0.185
	Cabbage-like intensity	0.173	0.167	0.245	0.475	0.058	0.633
	Skin intensity	0.138	**0.002**	**0.011**	0.496	0.540	0.166
	Sour intensity	0.232	0.053	0.544	0.376	0.371	0.207
	Kokumi intensity	0.120	0.650	0.171	0.928	0.733	0.601
	Sweet intensity	0.157	0.701	**0.007**	0.222	0.989	0.841
	General intensity	**0.028**	**0.001**	0.050	0.201	0.074	0.853
Taste/ aroma	Cabbage-like	0.806	0.232	**0.023**	0.692	**0.005**	0.281
	Chicken	**0.001**	**0.001**	0.360	0.280	**0.001**	0.792
	Sweet	0.676	0.848	**0.001**	0.755	0.844	0.261
	Salty	**0.001**	**0.031**	**0.001**	0.439	**0.003**	**0.038**
	Sour	0.340	0.905	0.317	0.464	**0.003**	0.148
	Umami	0.119	**0.008**	**0.001**	0.653	0.244	**0.002**
Aftertaste	Cabbage-like	0.101	0.147	0.210	0.655	**0.002**	0.226
	Fatty	**0.001**	**0.035**	0.258	0.637	**0.039**	0.933
	Umami	**0.001**	0.127	**0.001**	0.418	0.134	**0.010**

The bold marked values are the ones that are significant.

## Appendix

Table A1,Table A2
